# Artificial Intelligence in Hepatocellular Carcinoma: Current Applications, Clinical Performance, and Barriers to Implementation

**DOI:** 10.3390/jcm15072484

**Published:** 2026-03-24

**Authors:** Sri Harsha Boppana, Aditya Chandrashekar, Gautam Maddineni, Raja Chandra Chakinala, Ritwik Raj, Rohin B. Shivaprakash, Pradeep Yarra, Venkata C. K. Sunkesula, C. David Mintz

**Affiliations:** 1 Nassau University Medical Center, East Meadow, NY 11554, USA; 2 Department of Gastroenterology, Johns Hopkins Medicine, Baltimore, MD 21287, USA; adityac002@gmail.com; 3 School of Medicine, Creighton University, Omaha, NE 68124, USA; 4 Guthrie Robert Packer Hospital, Guthrie, PA 18840, USA; 5 National Institutes of Health, Baltimore, MD 21224, USA; 6 St. John’s Medical College Hospital, Bengaluru 560034, Karnataka, India; 7 The University of Texas Health Science Center at San Antonio, San Antonio, TX 78229, USA; yarra@uthscsa.edu; 8 MetroHealth Hospital, Cleveland, OH 44109, USA; kumarsvc@gmail.com; 9 Department of Anesthesiology & Critical Care Medicine, Johns Hopkins Medicine, Baltimore, MD 21287, USA

**Keywords:** hepatocellular carcinoma, artificial intelligence, machine learning, deep learning, radiomics, liver imaging, ultrasound, computed tomography, magnetic resonance imaging, pathology, risk stratification, surveillance, prognostication, treatment response prediction, external validation

## Abstract

Hepatocellular carcinoma (HCC) remains a major cause of cancer-related mortality worldwide, and its management is limited by heterogeneous risk profiles, suboptimal surveillance performance, diagnostic uncertainty in chronically diseased livers, and difficulty individualizing prognosis after treatment. The aim of this narrative review was to critically evaluate artificial intelligence (AI) applications across the HCC care continuum, with emphasis on their intended clinical role, reported performance, evidence maturity, and barriers to implementation. A major strength of this review is that it moves beyond a descriptive catalog of models by structuring the literature around clinically relevant decision points and by explicitly distinguishing emerging proof-of-concept tools from applications with stronger translational potential. Across risk stratification, surveillance, imaging-based diagnosis, pathology, treatment-response prediction, and prognostication, we found that AI consistently demonstrates promise, particularly for identifying patients at higher future HCC risk, improving lesion detection and characterization on ultrasound, CT, MRI, and contrast-enhanced ultrasound, assisting histopathologic classification, and predicting outcomes such as microvascular invasion, recurrence, survival, and response to locoregional therapies. However, we also found that the evidence base remains highly uneven: many diagnostic studies are retrospective and lesion-enriched rather than embedded in true surveillance populations, many prognostic models lack robust external validation and calibration assessment, and reference standards, imaging protocols, and dataset composition vary substantially across studies. These findings are clinically relevant because they highlight both where AI may offer near-term value and why most published systems are not yet ready for routine use. Overall, AI in HCC should be viewed as a rapidly evolving but still transitional field. Its future impact will depend not only on higher-performing algorithms but on clearly defined clinical use cases, multicenter and prospective validation, transparent reporting, workflow-aware evaluation, and implementation strategies that support safe, equitable, and scalable adoption.

## 1. Introduction

Hepatocellular carcinoma (HCC) accounts for most primary liver cancers and remains a leading cause of cancer-related death worldwide [1,2]. In 2022, an estimated 684,659 new cases and 597,434 deaths were attributed to HCC, with marked geographic variation in burden and risk-factor attribution [2]. Although hepatitis B virus and hepatitis C virus remain dominant drivers in many regions, metabolic risk factors are contributing increasingly to incident disease, making the population at risk for HCC progressively more heterogeneous [2].

This heterogeneity complicates the entire HCC care continuum, from selecting patients for surveillance to characterizing detected lesions, staging disease, guiding treatment, and monitoring for recurrence. Current AASLD and EASL guidance places HCC management within a structured pathway that includes surveillance of at-risk populations, recall imaging for suspicious findings, standardized diagnostic interpretation, multidisciplinary staging, and treatment selection according to tumor burden, liver function, and performance status [1,3]. Yet each step remains imperfect in routine practice, particularly when surveillance quality is suboptimal, lesions are indeterminate, or underlying liver disease complicates interpretation.

Artificial intelligence (AI) has therefore emerged as a potential adjunct across multiple HCC decision points, including risk stratification, surveillance support, imaging characterization, digital pathology, treatment-response prediction, and prognostication. At the same time, the evidence base is highly heterogeneous in both quality and clinical maturity. Many imaging studies are retrospective and lesion-enriched rather than embedded in true surveillance populations, whereas many prediction models report encouraging discrimination without equally rigorous evidence for calibration, external validity, or transportability across institutions, etiologies, scanners, and workflows [4,5,6,7,8].

The HCC AI literature is also difficult to interpret because it combines fundamentally different tasks under a single label. A model that predicts future HCC risk from longitudinal clinical data is methodologically distinct from a convolutional neural network that classifies MRI-detected lesions, a pathology model that infers molecular alterations from whole-slide images, or a radiomics signature that predicts recurrence after resection. The evidentiary standards for these applications differ as well: diagnostic studies are better interpreted using STARD-AI, whereas prediction and prognostic studies are better judged against TRIPOD + AI and PROBAST + AI [4,5,6].

The objective of this review is to critically evaluate AI applications across the HCC care continuum, with emphasis on their intended clinical role, reported performance, level of validation, and barriers to implementation. Rather than providing a purely descriptive catalog of models, we aim to distinguish between proof-of-concept systems, emerging but incompletely validated tools, and applications with stronger translational potential.

**Figure 1:**
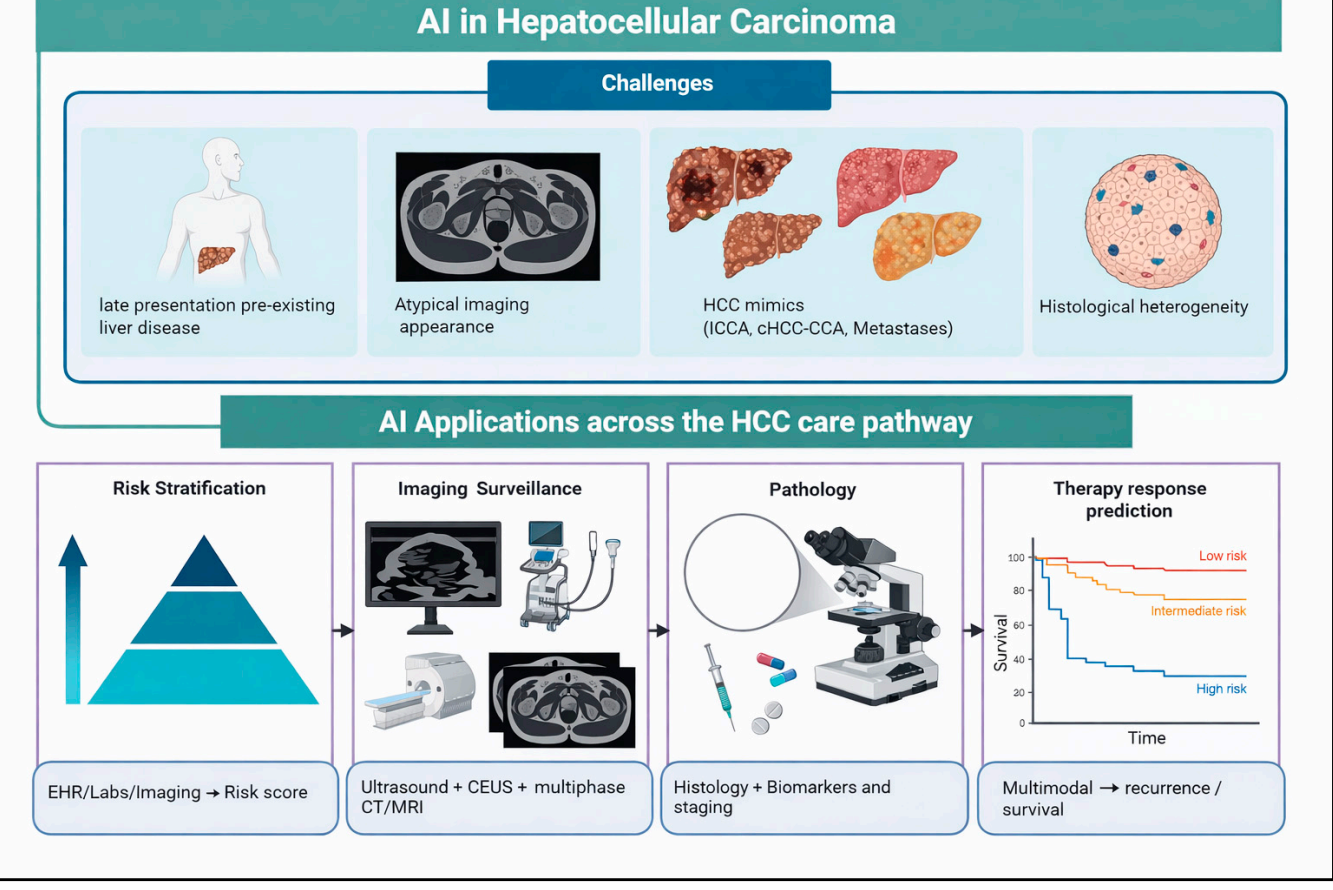
Overview of clinical challenges and artificial intelligence applications across the hepatocellular carcinoma care pathway. The upper panel depicts the four principal challenges facing AI development in HCC: late presentation in the setting of pre-existing chronic liver disease, atypical imaging appearance, imaging mimics including intrahepatic cholangiocarcinoma, combined hepatocellular-cholangiocarcinoma, and metastases, and histological heterogeneity. The lower panel maps the four major AI application domains: risk stratification integrating EHR, laboratory, and imaging inputs to generate individualized risk scores; imaging surveillance across ultrasound, CEUS, and multiphase CT and MRI for lesion detection and characterization; pathology AI incorporating histology and biomarkers for tumor classification, molecular inference, and staging; and therapy response prediction using multimodal inputs to estimate recurrence and survival across clinically meaningful risk strata. Abbreviations: AI, artificial intelligence; CEUS, contrast-enhanced ultrasound; cHCC-CCA, combined hepatocellular-cholangiocarcinoma; CT, computed tomography; EHR, electronic health record; HCC, hepatocellular carcinoma; iCCA, intrahepatic cholangiocarcinoma; MRI, magnetic resonance imaging.

To achieve this objective, we review AI applications in risk stratification and pre-malignant disease detection, imaging-based surveillance and diagnosis, pathology and biologic inference, treatment-response prediction, and prognostication. We then synthesize the quality and limitations of the current evidence, discuss implementation and regulatory barriers, and outline the steps needed to move HCC AI from promising retrospective models toward prospectively validated, clinically deployable tools [4,5,6,7,8]. Table 1 summarizes the evidence maturity of representative AI applications across the HCC care continuum, and Figure 1 provides a high-level overview of the major clinical challenges and AI application areas that frame the sections that follow.

## 2. Methods of Evidence Synthesis

We conducted a targeted narrative synthesis of the peer-reviewed literature describing AI applications across the HCC care continuum. Literature was identified through targeted searches of PubMed/MEDLINE, Scopus, Web of Science, and IEEE Xplore using combinations of terms related to hepatocellular carcinoma, artificial intelligence, machine learning, deep learning, radiomics, ultrasound, CT, MRI, pathology, treatment response, recurrence, and prognosis. The core search window emphasized studies published from 2015 onward to capture the contemporary deep-learning era, although older landmark studies were retained when they provided foundational context for methods that remain relevant to current HCC AI applications.

Peer-reviewed human studies in English were prioritized. Full-text articles were favored whenever available, whereas conference abstracts or brief reports were used sparingly and only when they represented an emerging HCC-specific application not yet supported by more complete published evidence. The review was organized around clinically relevant decision points in the HCC care continuum, including risk stratification and surveillance eligibility, imaging-based surveillance and diagnosis, pathology and biologic inference, treatment response prediction, and prognosis. Current guideline documents were also used to anchor the clinical workflow and contextualize the intended use of AI at each step [1,3].

Because the literature spans both diagnostic and prognostic study types, evidence was interpreted using reporting and appraisal frameworks matched to the underlying study design. Diagnostic imaging and pathology studies were read in light of STARD-AI [4]. Because the final checklist was published in 2025, it is applied here as a prospective standard for what future HCC AI studies should report, not as a retrospective indictment of earlier work. Prediction and prognostic studies were interpreted using TRIPOD + AI and PROBAST + AI, with emphasis on intended use, missing-data handling, discrimination, calibration, leakage control, external validation, and transportability [5,6]. Studies evaluating AI as an intervention that could alter clinician behavior or workflow were considered within the logic of CONSORT-AI and SPIRIT-AI, although truly prospective interventional evidence remains limited in HCC [7,8].

Consistent with the aims of this review, the synthesis is intentionally critical rather than purely descriptive. Reported performance metrics are discussed together with the underlying dataset type, reference standard, validation strategy, and remaining translational barriers so that retrospective proof-of-concept work is distinguished from clinically credible, externally evaluated applications. Table 2 provides a structured summary of representative AI models discussed in this review, organized by clinical task and including dataset size, modality, AI architecture, reference standard, reported performance metrics, external validation status, and key limitations as appraised through the frameworks described above.

## 3. Core AI Concepts Relevant to HCC

### 3.1. Artificial Intelligence, Machine Learning, and Deep Learning

Artificial intelligence (AI) is a broad term for computational systems that perform tasks such as classification, prediction, and pattern recognition, whereas machine learning (ML) refers to models that learn these relationships from data rather than from fixed hand-coded rules [54]. Deep learning is a subset of ML that uses multilayer neural networks, and convolutional neural networks (CNNs) are particularly effective for image analysis [54]. In this review, structured-data ML is most relevant to HCC risk stratification and surveillance eligibility, whereas deep learning is more prominent in ultrasound, CT, MRI, and digital pathology studies.

### 3.2. Convolutional Neural Networks and Radiomics

CNNs learn image features directly from pixels and therefore dominate many HCC applications in lesion detection, imaging classification, and slide-based pathology [54]. Radiomics, in contrast, refers to the high-throughput extraction of engineered quantitative features such as shape, intensity, and texture from medical images before downstream modeling [32]. This distinction is important for the later sections of this manuscript because many CT/MRI prognosis and recurrence studies use radiomics pipelines, whereas many ultrasound and pathology studies use CNN-based feature learning [32].

### 3.3. Validation Concepts Relevant to HCC AI Studies

The later sections of this review interpret HCC AI studies according to their intended clinical role and validation strategy, not by headline accuracy alone. For diagnostic studies in imaging or pathology, STARD-AI emphasizes transparent reporting of dataset construction, reference standards, algorithm evaluation, and applicability, which is especially important because many HCC imaging studies use retrospective, lesion-enriched datasets rather than true surveillance populations [4]. For prediction and prognostic studies, TRIPOD + AI and PROBAST + AI emphasize intended use, missing-data handling, discrimination, calibration, risk of bias, and external validation [5,6].

## 4. HCC Risk Factors and Pre-Malignant Disease Detection

### 4.1. Underlying Chronic Liver Disease and Screening Opportunity

Risk prediction is one of the most clinically attractive AI use cases in HCC because it could refine who undergoes surveillance, how often surveillance is performed, and when alternative modalities might be preferable. Current AASLD guidance recommends semiannual surveillance with ultrasound plus AFP for most patients with cirrhosis and selected patients with chronic hepatitis B, while not recommending routine surveillance for all patients with noncirrhotic advanced fibrosis after HCV cure or with MASLD alone [3]. These diseases develop gradually, making it possible to identify high-risk individuals throughout a screening period.

### 4.2. NAFLD and NASH

The NAFLD/NASH literature is relevant because metabolic liver disease is now a major driver of future HCC burden, but much of the AI work remains several steps removed from actual cancer prediction [3]. Early studies suggested that machine learning could identify metabolic signatures associated with NAFLD progression and NASH biology before overt malignancy [55]. These studies were biologically interesting, but they were not surveillance-allocation models and did not use future HCC as the endpoint. As such, they should be viewed as mechanistic or staging work rather than evidence that AI can already identify premalignant disease in a clinically deployable way.

A more clinically relevant example is the NAFLD ridge-score study by Yip et al., which used a screening cohort of 922 participants and defined NAFLD using proton magnetic resonance spectroscopy. The model achieved a validation AUROC of 0.88, but validation was internal only, and the task was NAFLD detection rather than future HCC risk, limiting its direct relevance to surveillance allocation [30].

More advanced NAFLD work has targeted fibrosis stages that are closer to the HCC pathway. In a multicenter U.S. biopsy-based cohort of 1370 patients with NAFLD, Chang et al. evaluated logistic regression, random forest, and artificial neural network models against histologic fibrosis stage; the random forest model achieved AUCs of 0.86 for at least F2 fibrosis and 0.89 for at least F3/F4 fibrosis, but evaluation was based on an internal 80/20 split rather than external or prospective validation [31]. The study is stronger than many small retrospective reports because of its multicenter design and biopsy anchor, yet it still does not establish surveillance eligibility for HCC, and would not satisfy current TRIPOD + AI expectations for clinically consequential prediction modeling.

### 4.3. Viral Hepatitis, Cirrhosis, and Fibrosis Progression Models

The chronic viral hepatitis literature is more directly tied to HCC risk and has produced some of the field’s earliest externally evaluated models. Singal et al. developed a machine-learning model in 442 patients with Child A or B cirrhosis and tested it in an independent cohort; external discrimination was modest, with a c-statistic of 0.64, but the study remains important because it included true external validation at a time when most models did not [9].

Konerman et al. approached the problem from a fibrosis-progression perspective using 72,683 patients with chronic hepatitis C from the Veterans Health Administration. The boosted-survival-tree approach achieved a concordance index of 0.774, but the study used a surrogate cirrhosis endpoint based on repeated APRI values and a predominantly male veteran cohort, limiting transportability beyond that setting [32].

A more surveillance-relevant example is the CirVir/Hepather work on personalized HCC surveillance in compensated HCV-related cirrhosis. In this true surveillance population undergoing semiannual ultrasound, random survival forest models achieved c-indexes of approximately 0.71 before sustained virologic response and 0.70 after sustained virologic response, outperforming simpler benchmark approaches; however, applicability to contemporary MASLD-dominant surveillance populations remains uncertain [11].

### 4.4. Evolving Model Performance and Multivariable Feature Integration

A recent comparative analysis across three prospective surveillance cohorts, including 4867 patients with cirrhosis, found that single-tree, random-forest, and deep-neural-network approaches did not significantly outperform established regression-based scores such as aMAP and FASTRAK [42]. Importantly, this was a true surveillance setting in which all patients underwent semiannual ultrasound rather than a retrospective enriched case set. The authors reported not only discrimination but also Brier score, decision-curve analysis, and calibration, and they found broadly similar predictive performance across model classes. That is a more credible and useful message for this review: ML may reveal clinically interpretable risk subgroups without necessarily producing a decisive gain in headline metrics. Accordingly, the emerging direction in this field is not simply larger models with more variables, but better-specified models with clearer intended use. The next generation of HCC risk models should be judged less by whether the AUC is marginally higher and more by whether the model is specified for a real decision.

## 5. Pathology

### 5.1. Overview and Role of AI in Liver Tumor Pathology

AI has found significant applications in pathology by enhancing lesion characterization and differentiation through precise image analysis of biopsy and resection samples. Early computational pathology studies showed that grading information can be extracted from morphology alone: Atupelage et al. reported a 95.97% five-class classification rate using liver-cell nuclear features, and another related multifractal-texture study achieved an average correct classification rate of about 95% across non-neoplastic tissue and Edmondson-Steiner grades [41,42]. These results are technically impressive, but they were based on retrospective regions of interest rather than whole-slide clinical workflows, so their real-world transportability is uncertain.

More recent whole-slide approaches have focused on clinically relevant classification tasks. Liao et al. distinguished HCC from adjacent normal tissue with an AUC of 0.988 in the test set and 0.886 in an external validation set, while also deriving a prognostic model after resection; however, this remained a retrospective digital-slide study using resection material rather than a prospective diagnostic workflow [44]. As a result, these models should be viewed as promising decision-support tools rather than deployment-ready pathology systems.

### 5.2. Emerging “Lab-Free” and Real-Time Approaches

Pathology AI has also expanded into “lab-free” or accelerated workflows intended to reduce reliance on conventional frozen-section processing. Using hyperspectral imaging of HCC tissue, Wang et al. reported sensitivity 0.871, specificity 0.888, and AUC 0.950 with a 1-D convolutional neural network, suggesting that optical spectral data may support rapid tumor identification [45]. Similarly, Lin et al. combined multiphoton microscopy with a VGG-16-based deep learning framework and achieved >90% accuracy in classifying HCC differentiation grade [46].

These studies are attractive because they point toward faster, less labor-intensive pathology workflows, but they remain highly experimental. Both relied on specialized imaging platforms, retrospective image sets, and narrow laboratory settings rather than routine pathology practice. They are better framed as proof-of-concept technologies than near-term clinical substitutes for standard histopathology.

### 5.3. Clinical Need and Diagnostic Variability

Pathology remains important in HCC because morphology carries information that imaging alone cannot fully resolve, particularly in atypical tumors, mixed phenotypes, and biologically aggressive subtypes. Calderaro et al. showed in 343 resected HCCs that histologic phenotypes were tightly linked to molecular classes: CTNNB1-mutated tumors were typically well differentiated and cholestatic, whereas TP53-mutated tumors were more often poorly differentiated, pleomorphic, and vascular invasive [56]. The same work identified the macrotrabecular-massive subtype as a pathologically and biologically distinct aggressive phenotype, a finding later reinforced by Ziol et al., who showed that this subtype accounted for about 12% of curatively treated HCCs and independently predicted early and overall recurrence after resection or ablation [57].

The need for diagnostic support is reinforced by human variability. In the Kiani study, unassisted accuracy differed meaningfully by experience level, from 0.946 in GI specialists to 0.842 in non-GI specialists and 0.858 in trainees. That variability makes pathology a natural target for AI augmentation, but it also means that pathology-AI tools should be tested not only for stand-alone performance, but also for how they influence users with different levels of expertise [43].

### 5.4. Digital Slide CNN Models and Human-AI Collaboration

The most clinically informative pathology-AI study in this section is the human-AI interaction experiment by Kiani et al. [43]. Their deep-learning assistant differentiated HCC from cholangiocarcinoma with model accuracies of 0.885 on a validation set of 26 whole-slide images and 0.842 on an independent test set of 80 whole-slide images. Across all 11 pathologists, assistance increased mean accuracy only modestly, from 0.898 to 0.914, and not significantly overall; however, it significantly improved performance in the subset of nine pathologists with defined experience levels (OR 1.499, *p* = 0.045). Crucially, when the model was correct, assistance increased the odds of a correct diagnosis (OR 4.289), but when the model was wrong, it substantially reduced pathologist accuracy (OR 0.253). This is one of the clearest demonstrations in liver pathology that AI can both help and harm, depending on how it is integrated into human decision-making. The central question is not whether CNNs can classify digital slides, because they clearly can, but whether they improve final diagnoses safely. Most pathology studies remain retrospective and slide-based, with limited prospective evaluation of user behavior, confidence thresholds, escalation rules, or failure modes. For that reason, pathology-AI tools should currently be framed as assistive systems, not autonomous classifiers.

### 5.5. Prediction of Molecular Alterations and Prognosis from Histology

A major advance in digital pathology is the ability to infer molecular biology from routine histology. In HCC, Chen et al. used an Inception-v3 framework on histopathology images and reported 96.0% accuracy for benign-versus-malignant classification and 89.6% accuracy for tumor differentiation, while also predicting several recurrent mutations [47]. In external validation, mutation prediction AUCs ranged from 0.71 to 0.89 for genes including CTNNB1, FMN2, TP53, and ZFX4 [48]. This is important because it moves pathology AI beyond morphology into genotype inference, but the model was still developed on retrospective slide datasets with internal and external validation cohorts derived from curated sources rather than real-time clinical workflows.

### 5.6. Toward Systemic Therapy Response Prediction

Using histology to infer systemic-therapy response is conceptually attractive but still immature in HCC. Outside liver cancer, deep learning has predicted immunotherapy-relevant biomarkers such as microsatellite instability directly from gastrointestinal histology, and pan-cancer studies have shown that routine H&E slides can capture clinically actionable molecular alterations [58,59,60].

In HCC itself, the strongest current evidence still comes from molecular rather than image-only biomarkers. In nivolumab-treated advanced HCC, Sangro et al. found that tumor PD-L1 positivity and a four-gene inflammatory signature were associated with improved response and survival, while Haber et al. reported an 11-gene interferon-activated signature associated with anti-PD1 benefit; these findings are biologically encouraging but do not yet establish a validated histology-only biomarker for routine treatment selection [60,61].

### 5.7. Limitations and Standardization Needs in Pathology AI

Pathology AI in HCC remains promising but methodologically fragile. Common limitations include small patient numbers, staining and scanner variability, sensitivity to annotation practice, and a near-complete lack of prospective validation. Future studies should standardize slide acquisition and preprocessing, report intended use clearly, and evaluate whether models remain robust when confronted with artifacts such as tissue folds, staining inconsistency, or out-of-distribution slides.

## 6. Radiomics

### 6.1. Ultrasound

#### 6.1.1. Guideline Context and Limitations

Ultrasound remains the backbone of HCC surveillance because it is inexpensive, widely available, and noninvasive. AASLD recommends ultrasound-based surveillance, typically with AFP, for at-risk patients, and the 2024 US LI-RADS update formalizes visualization scoring to account for suboptimal examinations in obesity, steatosis, and advanced cirrhosis [3,12]. Studies that ignore body habitus, hepatic steatosis, cirrhotic nodularity, operator dependence, and incomplete visualization risk overstating transportability [4,12].

#### 6.1.2. Deep Learning for Benign vs. Malignant Lesions

Schmauch and colleagues developed a supervised deep-learning model for focal liver lesion detection and characterization using 367 two-dimensional ultrasound images from 367 livers, followed by testing on a separate dataset of 177 patients [62]. The reported mean ROC-AUCs were 0.935 for lesion detection and 0.916 for lesion characterization, with 0.891 on the new test set across seven tasks. These results are promising, however the dataset was lesion-enriched rather than surveillance-based, and the task was lesion characterization rather than prospective surveillance detection.

A larger study by Tiyarattanachai et al. developed and validated a CNN using 40,397 retrospectively collected ultrasound images from 3487 patients, with external validation on 18,922 images from two additional hospitals. Internal-test detection was 87.0% with sensitivity 83.9% and specificity 97.1%, whereas external detection fell to 75.0% despite preserved sensitivity and specificity, illustrating how acquisition differences and case mix influence performance across institutions [13].

Another large multicentre retrospective diagnostic study (55 hospitals, 1052 patients with ≤3 cm lesions) developed an interpretable ML model (XGBoost with ultrasound features, radiomics, and clinical data) to classify small HCC [63]. The model showed excellent performance with internal validation AUC~0.934 and external AUC~0.899. However, selection bias persists because benign lesions that never undergo biopsy are underrepresented, and the case mix included many non-cirrhotic livers.

#### 6.1.3. Contrast-Enhanced Ultrasound and Pattern Recognition

AI applications in contrast-enhanced ultrasonography (CEUS) have mainly focused on post-detection lesion characterization rather than first-line surveillance. In a CEUS-based AI study for benign-versus-malignant focal liver lesions, the model achieved an AUC of 0.934 and 91.0% accuracy in the test set, and AI assistance improved radiologist sensitivity to 97.0–99.4%, with overall accuracy rising to 91.0–92.9% [33]. In a separate multicenter CEUS cine-clip study, computer-aided diagnosis achieved an AUC of 0.883 and 81.1% accuracy, and improved reader accuracy from 71.3% to 87.7% for inexperienced readers and from 80.9% to 90.3% for experienced readers [34]. More specifically, for indeterminate lesions, a multicenter retrospective study of CEUS LR-M nodules reported an internal test AUC of 0.796, sensitivity of 0.752, and specificity of 0.761, with external test AUCs ranging from 0.768 to 0.825; the model outperformed junior radiologists while performing comparably to senior readers [14]. However, these studies were retrospective, lesion-enriched diagnostic datasets rather than true surveillance cohorts, and the reference standard was lesion diagnosis rather than surveillance outcome. Their transportability is therefore limited by center-specific acquisition protocols, operator dependence, and the lack of standardized prospective CEUS workflows, so current evidence supports CEUS AI mainly as an adjunct for adjudicating already-detected indeterminate lesions rather than as a surveillance solution.

#### 6.1.4. Surveillance Performance and Early-Stage Detection Gap

The main reason ultrasound AI remains attractive is that baseline ultrasound performance for early-stage HCC is imperfect. In the large meta-analysis by Tzartzeva et al., the pooled sensitivity of ultrasound alone for any-stage HCC was 84%, but sensitivity for early-stage HCC fell to 47%; ultrasound plus AFP improved early-stage sensitivity to 63% [64]. These pooled studies were genuine surveillance cohorts rather than lesion-enriched diagnostic datasets, which makes them more clinically informative than many AI reports. At the same time, they also reveal the benchmark an AI system must beat under real surveillance conditions. An algorithm that reports an AUC above 0.90 in curated lesion datasets has not necessarily solved the true surveillance problem if it has not been tested in patients with subtle, subcentimeter, or poorly visualized lesions.

This limitation is reinforced by the emerging LI-RADS ultrasound visualization literature [62]. A recent meta-analysis found that visualization score C, representing severe limitations, was more common in patients with cirrhosis, NAFLD, and obesity [65]. That matters directly for AI transportability because the patients in whom surveillance most often fails are also those in whom model performance is least likely to generalize from clean retrospective datasets. A high-performing classifier cannot recover information that was never adequately captured on the original scan.

#### 6.1.5. Large-Scale CNN Training and AFP-Negative Detection

One appealing direction is targeted AI support for clinically difficult subgroups. In the AFP-negative HCC study by Zhang et al., an Xception-based model was developed using B-mode ultrasound images from surgically proven HCC and focal nodular hyperplasia cases in HBV-infected patients with focal liver lesions [35]. In the test cohort, the model achieved an AUC of 0.937, sensitivity of 96.08%, and specificity of 76.92%. These are strong diagnostic metrics, but the dataset was again lesion-enriched rather than surveillance-based, the validation strategy relied on internal splitting and cross-validation rather than external or prospective testing, and the reference standard was surgical pathology in a narrow comparator set. Its transportability is limited by HBV-specific case mix, use of pathology-confirmed lesions, and exclusion of the broader differential diagnosis encountered in everyday surveillance.

Even the larger external-validation work by Tiyarattanachai et al. should be interpreted similarly [13]. It shows that cross-site validation is feasible, but it still tests AI on known lesion images rather than on sequential surveillance examinations in which lesion prevalence is low and false positives carry downstream costs. The more defensible conclusion is that ultrasound AI has shown encouraging diagnostic assistance in lesion-rich settings, while evidence for actual surveillance deployment remains limited and should be judged against visualization quality, patient-level recall pathways, and prospective detection outcomes.

### 6.2. CT and MRI

#### 6.2.1. Indeterminate Lesions and the Need for Advanced Imaging

CT and MRI occupy a different place in the HCC pathway than ultrasound. They are primarily diagnostic and recall modalities, used after a positive or equivocal surveillance test or when ultrasound quality is inadequate, rather than universal first-line surveillance tools. AASLD guidance and LI-RADS frameworks reflect this distinction, and it is important for AI appraisal: many CT/MRI studies are diagnostic-classification studies in already detected lesions, not surveillance studies [3,12,66]. This distinction also explains why reported performance can appear stronger on CT and MRI than on ultrasound. Advanced cross-sectional imaging benefits from more standardized acquisition and clearer lesion depiction, but it is applied after clinical enrichment. That enrichment alone can inflate apparent performance relative to first-line surveillance.

#### 6.2.2. CT-Based Models and Radiomics Signatures

Early work in cross-sectional liver AI showed technical promise but also illustrated the gap between diagnostic assistance and clinically transportable HCC tools. In a retrospective PET/CT study of 98 consecutive patients who also underwent liver MRI within 2 months, Preis et al. reported neural-network AUCs of 0.905 and 0.896, compared with 0.786 and 0.796 for blinded expert readers; after access to network output, reader AUCs improved to 0.924 and 0.881 [36]. This was not a surveillance dataset and was not HCC-specific; rather, it was a diagnostic adjunct study using expert MRI interpretation as the reference standard, so its applicability to HCC surveillance or CT-based lesion characterization is limited.

Among HCC-focused CT radiomics studies, Mokrane et al. provide a more clinically relevant but also more sobering example. In a multicenter retrospective cohort of 178 cirrhotic patients from 27 institutions with biopsy-proven indeterminate nodules, the radiomics signature achieved an AUC of 0.70 in the discovery cohort and 0.66 in the validation cohort [15]. The strength of this study is its use of a difficult, real diagnostic subgroup and a histologic reference standard; however, the validation sample was small (n = 36), performance was only modest, and the lesion-enriched design differs substantially from a true surveillance population.

Deep-learning CT classification studies have generally reported higher performance, but most remain retrospective and internally validated. Yasaka et al. trained a CNN on augmented dynamic contrast-enhanced CT image sets derived from 460 patients and tested it on 100 liver mass image sets obtained later at the same institution, reporting a median accuracy of 0.84 and a median AUC of 0.92 for differentiating malignant from nonmalignant lesion categories [37]. Similarly, Shi et al. retrospectively analyzed 342 patients with 449 focal liver lesions and found test-set AUCs of 0.925 for a four-phase protocol, 0.862 for a three-phase protocol without portal venous phase, and 0.920 for a three-phase protocol without precontrast imaging, with corresponding accuracies of 83.3%, 81.1%, and 85.6%, respectively [38]. In both studies, the datasets were lesion-enriched diagnostic cohorts rather than surveillance populations, validation was effectively internal or single-center temporal testing, and the reference standard was lesion diagnosis or best available clinical diagnosis rather than prospective surveillance outcome. Their transportability therefore remains uncertain because case mix, contrast timing, scanner protocols, and prevalence of indeterminate lesions vary substantially across institutions.

Taken together, these CT studies support AI as a potentially useful adjunct for lesion characterization and diagnostic workflow support, but not yet as evidence of deployable surveillance technology. Under a STARD-AI lens, the key limitation is not that performance is poor, but that most studies still rely on retrospective, lesion-enriched cohorts with limited external validation and narrow intended-use settings, making generalization to routine HCC surveillance or broad multi-center practice uncertain.

#### 6.2.3. CT Surveillance Performance and AI-Augmented Reading

CT is not a routine first-line surveillance tool for most at-risk patients, and older data help explain why. In cirrhotic explant and resection specimens, triple-phase helical CT detected only 8 of 76 dysplastic nodules (10%), including 14% of high-grade and 7% of low-grade dysplastic nodules, underscoring limited sensitivity for premalignant or very early lesions in cirrhotic livers [67]. Likewise, perfusion CT studies in early HCC have shown biologic plausibility rather than deployable detection performance; in 35 cirrhotic patients with histologically proven HCC 3 cm or smaller, tumor perfusion metrics differed significantly from background liver, but the study was small and physiologic rather than a diagnostic accuracy trial [68].

A meta-analysis of 40 studies found that MRI outperformed CT for HCC diagnosis, with pooled per-lesion sensitivity favoring MRI over multidetector CT (80% versus 68%), and both modalities performed worse for lesions under 1 cm [16]. More recent AI work suggests CT may still be useful as a recall or diagnostic-assistance modality once cross-sectional imaging is obtained. Wang et al. trained a deep-learning model on 7512 patients and achieved internal and external AUROCs of 0.887 and 0.883, respectively, but this was still a retrospective diagnostic cohort rather than a surveillance study [16]. Other retrospective lesion-enriched CT studies have reported similarly high discrimination, including a CNN with a median AUC of 0.92 for dynamic contrast-enhanced CT mass classification and deep-learning AUCs up to 0.925 using multiphase CT protocols, but these studies were performed in known lesion cohorts, relied on internal or single-center validation, and used lesion diagnosis rather than prospective surveillance outcome as the reference standard [38,69]. Accordingly, the most defensible conclusion is that CT-based AI currently appears more credible for diagnostic adjudication during recall imaging than for primary HCC surveillance, and its transportability remains limited by protocol variation, contrast timing, case-mix enrichment, and the persistent difficulty of very small lesions.

#### 6.2.4. Segmentation Automation and LiTS Challenge

Automatic segmentation is one of the more mature technical subfields in liver imaging AI. The LiTS benchmark, which used 131 training CT volumes and 70 unseen test scans from multiple institutions, reported best liver Dice scores up to 0.963 and tumor Dice scores improving from 0.674 in the earliest challenge phase to 0.739 in later iterations [70]. Because LiTS uses a public benchmark with hidden test data, it offers stronger methodological discipline than many single-center segmentation papers. Even so, the benchmark includes diverse primary and secondary liver tumors, not HCC alone, and benchmark performance does not automatically translate into clinical utility for HCC surveillance or diagnosis. Small lesions and low-contrast tumors remain substantially harder to segment than larger lesions. The key point is that segmentation accuracy is necessary but not sufficient. Under STARD-AI logic, a segmentation model must still be linked to a clinically relevant downstream use case, such as response assessment, volumetry, or workflow efficiency. Otherwise, excellent Dice scores risk being overinterpreted as evidence of diagnostic readiness. 

#### 6.2.5. MRI-Based Models and Practical Constraints

MRI already provides rich multiparametric lesion characterization in cirrhosis through T1- and T2-weighted imaging, diffusion-weighted imaging, dynamic contrast phases, and hepatobiliary phase techniques when available [36,71]. MRI-based AI should therefore be viewed as an adjunct for lesion adjudication after detection, not as a replacement for surveillance ultrasound or for structured radiologic frameworks such as LI-RADS [12]. Conventional MRI already performs strongly for HCC diagnosis, which means AI must demonstrate incremental value rather than simply report high discrimination in known-lesion datasets. Hamm et al. reported 92% accuracy, 92% sensitivity, and 98% specificity for a multiphasic MRI CNN in a 494-lesion proof-of-concept study, but the use case remained lesion classification after presentation rather than population-level surveillance [40].

MRI also faces practical constraints that are often underemphasized in AI manuscripts. Cost, scanner availability, protocol heterogeneity, contrast use, and workflow burden all shape real-world applicability. These issues are especially relevant when considering MRI as an alternative surveillance strategy in patients with poor ultrasound visualization, because diagnostic accuracy alone does not determine deployability [72,73].

#### 6.2.6. ML/DL Approaches to Tumor Differentiation on MRI

Radiomics and deep-learning studies have shown promising discrimination, but nearly all use retrospective, lesion-enriched datasets. In non-contrast 3D T1-weighted MRI, Oyama et al. analyzed 150 tumors comprising 50 HCCs, 50 metastases, and 50 hemangiomas and reported 92% accuracy for HCC versus metastasis and 90% accuracy for HCC versus hemangioma using texture analysis [39]. However, this was not a surveillance cohort, validation was internal, and the reference standard was tumor class assignment within a balanced retrospective dataset, so transportability to routine cirrhosis imaging is limited. Similarly, Oestmann et al. trained a 3D CNN on 150 pathologically proven lesions (93 HCC, 57 non-HCC) and reported 87.3% overall accuracy, AUC 0.912, 92.7% sensitivity, and 82.0% specificity for HCC classification [17]. The pathology-based reference standard is a strength, and inclusion of atypical lesions improves realism, but the study remained retrospective, used repeated internal subsampling rather than true external or prospective validation, and performance declined as lesions became more atypical, which is exactly where clinical decision support is most needed. A larger multicenter MRI deep-learning study has also shown that external validation is feasible, with external-test AUC 0.90, 87% sensitivity, and 93% specificity on hepatobiliary-phase images, but this too was a diagnostic lesion dataset rather than a surveillance population [74]. Overall, MRI-based AI appears most credible for post-detection lesion differentiation, while generalizability remains constrained by retrospective case enrichment, scanner and protocol heterogeneity, and the need to demonstrate incremental value beyond expert MRI interpretation and LI-RADS-based assessment.

#### 6.2.7. Multi-Input DL Systems and Interpretability

Zhen et al. developed a deep-learning system combining MRI and clinical data using 1210 patients for training and an external cohort of 201 patients for validation [74]. This is methodologically stronger than many earlier MRI studies because it includes an external dataset and explicitly tests multimodal integration. HCC and other liver cancers were classified with remarkable accuracy by the DL system, which achieved sensitivity and specificity on par with skilled radiologists. However, the dataset was still composed of patients with established liver tumors rather than a surveillance population, and the reference standard was tumor diagnosis in a specialty setting. The model therefore supports multimodal diagnostic assistance, but not surveillance implementation. Moreover, despite external validation, reporting on calibration and decision-threshold behavior is limited, and interpretability remains restricted relative to rule-based systems such as LI-RADS.

A newer multicenter non-contrast MRI study by the same group extends this concept across three centers and multiple internal and external datasets, again suggesting that AI can preserve reasonable lesion discrimination even without contrast [18]. That is clinically attractive for patients who cannot receive gadolinium. Still, the use case remains lesion diagnosis after presentation, not population-level surveillance, and transportability will depend on scanner variation, protocol harmonization, and prospective testing in real workflows.

#### 6.2.8. Radiomics Limitations Across Modalities

Across ultrasound, CT, and MRI, the dominant pattern is consistent: performance is usually strongest in retrospective, lesion-enriched datasets with internal or limited external validation, and weakest in the real-world scenarios that matter most clinically, including early-stage tumors, poor-quality ultrasound examinations, indeterminate lesions, and heterogeneous community practice. STARD-AI provides a useful framework here because it shifts attention from headline metrics to intended use, dataset provenance, reference-standard clarity, exclusions, and applicability. Many imaging-AI papers still fall short of this standard by providing insufficient detail on case selection, image quality, missingness, and workflow position.

For this reason, the central question is no longer whether radiomics and deep learning can classify liver lesions under selected conditions. They clearly can. The harder and more clinically relevant question is whether they improve surveillance or diagnostic pathways when prospectively embedded in care, across variable imaging quality, diverse etiologies, and shifting prevalence.

## 7. Prediction of Treatment Outcomes

### 7.1. Transarterial Chemoembolization

AI studies in TACE have focused on two distinct tasks: pre-treatment response prediction and post-treatment survival stratification. In a 36-patient MRI-plus-clinical cohort, Abajian et al. achieved 78% accuracy, 62.5% sensitivity, and 82.1% specificity for classifying responders versus nonresponders using qEASL as the reference standard [21]. In a separate 105-patient CT study, Morshid et al. improved predictive accuracy from 62.9% with BCLC stage alone to 74.2% using a machine-learning model, with ROC AUC 0.73 [49]. These studies established feasibility, but both were small, retrospective studies.

Larger imaging-based models report stronger discrimination but still arise from retrospective treatment cohorts. Liu et al. analyzed 130 patients undergoing their first TACE and reported a validation AUC of 0.93 for a CEUS-based deep-learning model using mRECIST response as the reference standard [22]. Peng et al. trained a ResNet50 model on pretreatment CT from 562 patients and reported AUCs of 0.95–0.97 across response categories, with external validation accuracies of 85.1% and 82.8% in two additional cohorts [23]. More recently, Dai et al. developed a multicenter repeat-TACE prognostic score in 310 patients from three hospitals and reported AUCs of approximately 0.97 in development, 0.89 in validation, and 0.76 to 0.84 in external cohorts, with calibration and decision-curve analysis also reported [24]. However, most TACE models remain retrospective, and many outcomes are radiologic surrogates rather than patient-centered endpoints, so true clinical usefulness still requires prospective evaluation.

Mähringer-Kunz et al. addressed a related question by predicting one-year survival after TACE using an artificial neural network. In this pilot retrospective cohort, internal validation yielded AUC 0.83 ± 0.06, sensitivity 77.8%, and specificity 81.0%, but the model was not externally validated and remained vulnerable to overfitting in a pilot-scale design [25].

### 7.2. Stereotactic Body Radiotherapy

AI work in SBRT has focused less on tumor response than on toxicity prediction. Ibragimov et al. developed a deep-learning framework using 125 liver SBRT cases, including 36 HCCs, and combined 3D dose-plan analysis with pretreatment clinical features [50]. The CNN alone achieved AUC 0.79, which improved to 0.85 when combined with fully connected networks for numerical features. This is clinically relevant because hepatobiliary toxicity can constrain treatment intensity, but the cohort was not HCC-specific, the endpoint was toxicity rather than oncologic benefit, and the study did not constitute external or prospective validation. The model is better viewed as a promising planning adjunct than a deployable HCC-specific toxicity tool. AI models have also been used to forecast the results of radiofrequency ablation (RFA) and stereotactic body radiation treatment (SBRT).

### 7.3. Radiofrequency Ablation

For radiofrequency ablation, Wu et al. developed ANN models to predict one-year and two-year disease-free survival after CT-guided percutaneous RFA. In the 252-patient one-year DFS cohort, internal validation yielded an accuracy of 85.0% and an AUC of 0.84, whereas a simulated prospective validation fell to an accuracy of 70.0% and an AUC of 0.77; for two-year DFS, performance declined to an AUC of 0.75 internally and 0.72 in simulated prospective testing [29].

### 7.4. Post-Resection Survival and Recurrence

Post-resection prediction has been one of the most active HCC AI domains. Saillard et al. used whole-slide histology to predict survival after resection, developing two deep-learning models in a 194-patient discovery cohort and validating them in an independent TCGA cohort of 328 patients [27]. In the discovery set, the pathologist-guided and fully automated models achieved c-indices of 0.78 and 0.75, respectively, and both retained higher discriminatory power than a baseline clinicopathologic score in the external TCGA set. This is one of the stronger studies because it included independent external validation and biologically interpretable poor-prognosis features such as vascular spaces, macrotrabecular architecture, and reduced immune infiltration.

Recurrence modeling after resection has also advanced beyond single-center signatures. Ji et al. assembled a multi-institutional CT cohort of 470 patients with solitary HCC and reported c-indices of 0.733 to 0.801 for combined preoperative and postoperative models, together with integrated Brier scores of 0.147 to 0.165 and explicit calibration assessment [26]. This is methodologically stronger than many earlier radiomics papers because it includes calibration, external validation, and decision-curve analysis. Nevertheless, it remains retrospective, depends on manual or semi-manual imaging workflows, which still leaves room for bias and limited cross-platform transportability. Xu et al. approached recurrence differently, using a Bayesian network with latent variables to distinguish early, late, and no recurrence after resection, but the study provided less transparent reporting of cohort splits and calibration than is now expected [53].

### 7.5. Biomarker and Guideline-Relevant Prediction

In order to better inform judgments regarding liver resection, AI has also been utilized to assess the predictive power of specific biomarkers. Feng et al. developed an MRI radiomics model using 110 training and 50 validation patients to predict microvascular invasion before hepatectomy, achieving a validation AUC of 0.83, 90.0% sensitivity, and 75.0% specificity [19]. This is clinically relevant because MVI influences resection strategy, transplant candidacy discussions, and recurrence risk. However, the endpoint was a surrogate biologic feature rather than a direct patient outcome.

A related line of work uses machine learning to refine guideline-relevant surgical selection. In an international multicenter cohort of 976 resected patients with BCLC-0, A, and B disease, Tsilimigras et al. used CART to identify the preoperative and postoperative variables most strongly associated with overall survival, supporting the idea that AI may help contextualize resection decisions beyond stage labels alone [20].

### 7.6. Liver Transplantation Outcomes

Prediction after liver transplantation remains comparatively underdeveloped but potentially important. Guo et al. used pretransplant CT radiomics to predict post-transplant recurrence-free survival and reported a combined radiomics-clinical nomogram with a c-index of 0.785 in the training cohort and 0.789 in validation, with calibration curves showing agreement in both sets [28]. However, the model was derived from a small retrospective cohort and lacked true external site validation.

## 8. Current Challenges in AI for HCC Risk Prediction and Prognostication

### 8.1. Need for Algorithm Standardization

A central limitation of the HCC AI literature is that apparently similar models are often developed for different targets, using different reference standards, case-selection rules, and validation frameworks, making head-to-head interpretation difficult. Diagnostic studies should now be judged against STARD-AI, and prediction or prognostic studies should be assessed using TRIPOD + AI and PROBAST + AI [4,5,6].

If future studies move beyond retrospective evaluation and test AI as an intervention that changes clinician behavior or patient management, protocols and trial reports should follow SPIRIT-AI, CONSORT-AI, and FUTURE-AI, which require explicit reporting of the clinical setting, human-AI interaction, input-output handling, and error analysis [7,8,75]. Table 3 summarizes the most common sources of bias in the HCC AI literature and pairs each with the minimum reporting expectations that future studies should meet.

### 8.2. Need for Data Sharing and Open-Source Algorithms

Progress in HCC AI will remain constrained unless datasets become larger, more diverse, and more auditable. STARD-AI explicitly encourages transparent reporting of dataset practices, public availability of data and code, and external audit or evaluation of model outputs, while FUTURE-AI identifies traceability, usability, robustness, and lifecycle monitoring as core requirements for trustworthy deployment [4,76]. Broader clinical research expectations have also evolved: the International Committee of Medical Journal Editors (ICMJE) requires data-sharing statements for clinical trial reports [77]; however, these policies do not by themselves ensure practical access to usable individual-participant data. In a post-policy evaluation of 487 trial reports published in JAMA, The Lancet, and NEJM, 68.6% stated that data would be shared, but only 0.6% had deidentified individual-participant datasets publicly available [78]. For HCC AI, the challenge is even greater because model development may require linked electronic health records, imaging archives, and digital whole-slide pathology images, all of which raise substantial issues of governance, de-identification, storage, linkage, and access control; whole-slide images, for example, may contain identifiable labels or metadata and can be linkable across datasets [79]. Accordingly, future HCC AI studies should emphasize dataset diversity, standardized handling of missing data, explicit reporting of how development cohorts differ from target clinical populations, and transparent access practices. The goal should be transportable and accountable models, not maximal data accumulation alone.

### 8.3. Need for Diverse Populations

Racial, cultural, and socioeconomic diversity in AI models for HCC prediction, diagnosis, and prognosis has typically been lacking. Since the validity and scope of input data establish how accurate AI algorithms are, this lack of diversity presents a serious problem. Future studies must validate AI technologies across a range of demographics, including members of racial and ethnic minorities and patients from various socioeconomic backgrounds. This emphasizes again how crucial data sharing between researchers and institutions is to building representative cohorts.

### 8.4. Examples from Other Disciplines

Experience from other disciplines shows that regulatory translation requires far more than strong retrospective accuracy. In January 2025, the FDA stated that it had authorized more than 1000 AI-enabled devices through established premarket pathways and noted that its public AI-enabled device list is not comprehensive and may in future identify devices using foundation models or large language models [80,81]. Importantly, the list is not comprehensive and is being expanded. Yet despite the volume of HCC AI publications, obvious HCC-specific, regulatory-labelled tools are not prominent on that list. Outside the United States, AI medical software is increasingly governed through formal regulatory pathways. In the European Union, medical AI software is regulated within the MDR and IVDR framework, with Medical Device Coordination Group guidance clarifying qualification and classification of software and newer guidance addressing the interaction between those rules and the EU AI Act [82]. In China, the National Medical Products Administration has also issued classification guidance for AI-based medical software products [83]. These frameworks matter because they emphasize lifecycle evidence, post-market obligations, and accountability rather than one-time retrospective performance claims. Table 3 outlines the major barriers to clinical implementation of artificial intelligence in hepatocellular carcinoma care and pairs each barrier with pragmatic mitigation strategies and typical stakeholders responsible for execution.

## 9. Future Research Directions

The next phase of HCC AI should prioritize multimodal, clinically targeted models that integrate longitudinal clinical data, imaging, pathology, and biomarkers around specific decisions such as surveillance escalation, lesion characterization, treatment selection, and recurrence monitoring, rather than continuing to generate isolated retrospective classifiers. To improve generalizability, future development should rely more on multi-institutional and federated approaches, especially as HCC populations become more heterogeneous across etiologies, imaging quality, and care settings [84,85]. Foundation models may further improve transferability, but in HCC, they should currently be viewed as enabling infrastructure rather than ready-for-use clinical tools [51].

**Figure 2:**
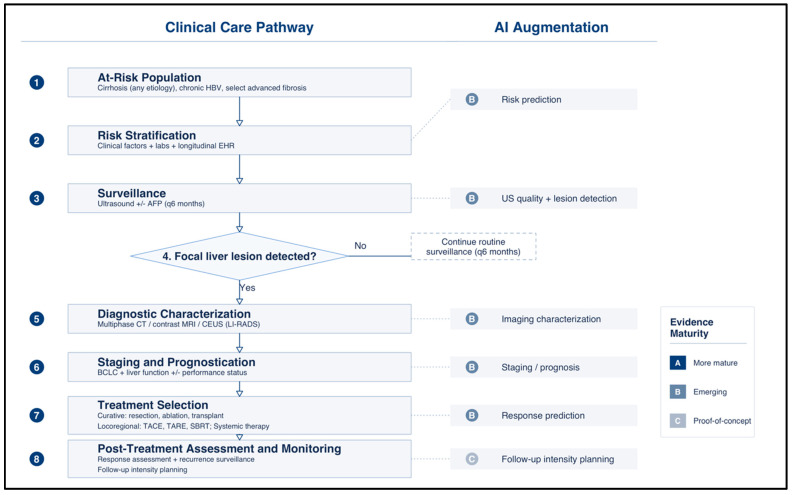
Artificial intelligence augmentation across the hepatocellular carcinoma clinical care pathway. The left column depicts the sequential clinical workflow for at-risk patients, beginning with identification of the at-risk population (cirrhosis of any etiology, chronic hepatitis B virus infection, or select patients with advanced fibrosis), proceeding through risk stratification using clinical factors, laboratory data, and longitudinal electronic health record inputs, and advancing to semiannual surveillance with ultrasound with or without alpha-fetoprotein. When no focal liver lesion is detected, patients continue routine surveillance at six-month intervals. When a focal lesion is identified, the pathway advances to diagnostic characterization with multiphase CT, contrast-enhanced MRI, or contrast-enhanced ultrasound interpreted using LI-RADS criteria, followed by staging and prognostication using the Barcelona Clinic Liver Cancer system with assessment of liver function and performance status, treatment selection across curative (resection, ablation, transplantation), locoregional (TACE, TARE, SBRT), and systemic therapy options, and finally post-treatment assessment and monitoring including response assessment and recurrence surveillance. The right column maps the corresponding AI augmentation opportunity at each step, with evidence maturity graded as more mature (A), emerging (B), or proof-of-concept (C). All current AI applications in HCC—spanning risk prediction, ultrasound quality assessment and lesion detection, imaging characterization, staging and prognosis, and treatment response prediction—are classified as emerging (B), while follow-up intensity planning remains at the proof-of-concept stage (C), reflecting the absence of prospectively validated, workflow-integrated tools across the entire continuum.

Just as importantly, the field needs a clearer evidence ladder. Retrospective internal validation should be treated as hypothesis-generating, multicenter external validation as the minimum threshold for strong claims, and prospective silent-deployment and interventional studies as the standard for testing clinical utility. Because HCC care pathways and surveillance populations are evolving, future systems will also require continuous monitoring for dataset shift, bias, and performance drift rather than one-time validation alone. The goal should not be the highest retrospective AUC, but transportable, accountable, and update-ready models that improve real-world HCC care. Figure 2 illustrates the sequential HCC clinical care pathway alongside the corresponding AI augmentation opportunity at each step, with evidence maturity graded to reflect the current state of validation from risk prediction through post-treatment monitoring.

## 10. Conclusions

Artificial intelligence has clear potential to augment multiple points along the HCC care continuum, including risk stratification, surveillance support, imaging characterization, digital pathology, treatment-response prediction, and post-treatment prognostication. However, the current evidence base remains uneven. Many diagnostic studies are still retrospective and lesion-enriched rather than embedded in true surveillance populations, while many prediction and prognostic models remain limited by incomplete external validation, inconsistent calibration reporting, and uncertain transportability across etiologies, institutions, scanners, and workflows. As a result, HCC AI should not be viewed as a single mature field but rather as a set of related applications at different stages of clinical readiness. The most important next step is not simply building larger models but generating stronger evidence. Future HCC AI studies should prioritize clearly defined clinical use cases, multicenter validation, transparent reporting, prospective workflow evaluation and ongoing monitoring for dataset shift, bias, and performance drift. At present, the gap between publication volume and real-world clinical integration remains substantial, particularly for HCC-specific tools. The goal, therefore, should not be maximal retrospective accuracy alone, but the development of transportable, accountable, and clinically useful systems that improve decision-making and patient outcomes in real-world HCC care.

## Figures and Tables

**Table 1:** Evidence maturity of representative AI applications across the HCC care continuum. For each workflow step, the table identifies representative studies cited in this review, the highest level of external validation achieved to date, and the key barriers that must be addressed before routine clinical implementation. Validation levels are assigned based on the most rigorous study design reported among the cited works for that domain; barriers reflect limitations common across studies rather than those of any single publication.

Workflow Step	Representative Studies/Tools Cited in Manuscript	Highest Level of Validation Achieved	Key Remaining Barriers Before Routine Use
Risk stratification	Singal 2013 [9]; Audureau 2020 [10]; Nahon 2026 [11]	External validation in independent cirrhosis cohorts; multicohort evaluation in prospective surveillance datasets	Modest discrimination in older models; limited evidence that AI-guided risk stratification changes surveillance modality, interval, stage at detection, or survival
Surveillance	Schmauch 2019 [12]; Tiyarattanachai 2021 [13]	Multicenter external validation in retrospective focal-lesion ultrasound datasets	Lesion-enriched rather than surveillance populations; operator dependence; visualization constraints in obesity, steatosis, and cirrhosis; limited prospective testing
Diagnostic imaging characterization	Xiao 2024 [14]; Mokrane 2020 [15]; Wang 2021 [16]; Oestmann 2021 [17]; Zhen 2025 [18]	Multicenter or external validation in retrospective diagnostic cohorts	Spectrum bias, scanner and protocol heterogeneity, limited testing in very small or truly indeterminate lesions, and little prospective workflow evidence
Staging/prognostication	Feng 2019 [19]; Tsilimigras 2020 [20]	Separate validation cohort for MRI-based microvascular invasion prediction; international multicenter retrospective survival stratification	Some endpoints are surrogate biologic markers; limited calibration-focused reporting; no prospective trials showing improved treatment allocation
Treatment selection	Abajian 2018 [21]; Liu 2020 [22]; Peng 2020 [23]; Dai 2025 [24]; Mähringer-Kunz 2020 [25]	External validation in retrospective TACE cohorts; more recent multicenter retrospective external testing for repeat-TACE prognosis	Heavy reliance on retrospective imaging cohorts and surrogate radiologic endpoints; limited prospective decision-impact evidence; uncertain thresholding for treatment change
Post-treatment monitoring	Ji 2019 [26]; Saillard 2020 [27]; Guo 2019 [28]; Wu 2017 [29]	Multi-institutional external validation for recurrence and independent external validation for histology-based survival prediction	Retrospective design predominates; populations differ across resection, ablation, and transplant settings; no prospective evidence that model-guided follow-up improves outcomes

Abbreviations: AI, artificial intelligence; MRI, magnetic resonance imaging; TACE, transarterial chemoembolization.

**Table 2:** Representative AI Models in HC.

First Author, Year	Clinical Task	Dataset Size (Development/Validation)	Modality/Input	AI Architecture	Reference Standard	Reported Metrics	External Validation	Key Limitations
Yip, 2017 [30]	NAFLD detection/pre-surveillance triage	922 total (random training/validation split)	Clinical + lab	Ridge regression/elastic net feature selection	Proton-MRS NAFLD	AUROC 0.87 train; 0.88 validation	No	General-population NAFLD model, not HCC endpoint; single health-system cohort
Chang, 2023 [31]	Fibrosis staging in NAFLD	1370 total (80% train/20% test)	Clinical + lab + FibroScan	LR, RF, ANN	Histologic fibrosis stage	RF AUC 0.86 (≥F2), 0.89 (≥F3), 0.89 (F4)	No	Biopsy-based staging cohort rather than surveillance cohort; internal split only
Singal, 2013 [9]	HCC risk prediction in cirrhosis	442 derivation + independent HALT-C validation cohort	Clinical variables	Machine-learning risk model	Incident HCC during follow-up	Validation c-statistic 0.64 vs. 0.61 for regression	Yes	Modest discrimination; older Child A/B cirrhosis population
Konerman, 2019 [32]	Cirrhosis progression prediction in chronic hepatitis C	72,683 total	Longitudinal EHR clinical + lab	Boosted survival tree	Repeated APRI >2 as cirrhosis proxy	Concordance index 0.774	No	Surrogate cirrhosis endpoint rather than HCC; predominantly male VHA population
Audureau, 2020 [10]	Personalized HCC surveillance in HCV cirrhosis	836 derivation/668 external validation	Surveillance clinical data	Fine-Gray, decision tree, random survival forest	Incident HCC during semiannual ultrasound surveillance	RSF C-index 0.71 pre-SVR; 0.70 post-SVR	Yes	Restricted to compensated biopsy-proven HCV cirrhosis; uncertain transportability to MASLD/HBV
Nahon, 2026 [11]	HCC risk stratification during surveillance	4867 total (random training/validation split)	Clinical + lab surveillance data	Single tree, RF, DNN survival models	Incident HCC during surveillance	No significant gain over aMAP/FASTRAK; Brier score and calibration reported	Yes	No interventional deployment; complex models did not clearly outperform simpler benchmarks
Schmauch, 2019 [12]	Focal liver lesion detection/characterization	367 training images/177-patient test set	US still images	Supervised-attention deep learning	Annotated lesion labels	AUC 0.935 detection; 0.916 characterization; 0.891 on new test set	Independent test set	Small lesion-enriched still-image dataset, not surveillance
Tiyarattanachai, 2021 [13]	Focal liver lesion detection/diagnosis	3487 patients; 40,397 dev images/6191 internal-test images/18,922 external-val images	US still images	CNN	Labeled lesion diagnosis	Internal detection 87.0%, sensitivity 83.9%, specificity 97.1%; external detection 75.0%, sensitivity 84.9%, specificity 97.1%	Yes	Known-lesion dataset rather than surveillance population; performance drift across sites
Hu, 2021 [33]	Benign vs. malignant focal liver lesion classification	363 train/211 test patients	CEUS videos + clinical data	Composite deep-learning model	Final benign vs. malignant lesion diagnosis	Test AUC 0.934; accuracy 91.0%; AI-assisted reader sensitivity 97.0–99.4%	No	Single-center retrospective lesion-characterization study
Ta, 2018 [34]	Benign vs. malignant focal liver lesions	106 subjects/105 analyzed cine clips	CEUS cine clips	ANN + SVM CAD	Histology, CE-CT/MRI, and/or ≥6 mo follow-up	CAD classified 95/105 clips; accuracy 81.1%; AUC 0.883	Multicenter data, no external holdout	Retrospective lesion-characterization study; cine quality exclusions and expert-center acquisition
Xiao, 2024 [14]	HCC vs. non-HCC among CEUS LR-M nodules	168 internal cohort/110 external-test patients	Dynamic CEUS quantitative parameters	Random forest	Final lesion diagnosis for LR-M nodules	Internal-test AUC 0.796; sensitivity 0.752; specificity 0.761; external AUC 0.768–0.825	Yes	Restricted to LR-M nodules; retrospective tertiary-center CEUS workflow
Zhang, 2022 [35]	AFP-negative HBV-related HCC vs. FNH	407 cases/413 lesions; model cohort 305 cases/test cohort 102 cases	B-mode US	Xception CNN	Surgically proven lesion diagnosis	AUC 93.68%; sensitivity 96.08%; specificity 76.92%; accuracy 86.41%	No	HBV-specific, surgically proven lesion set with narrow comparator spectrum
Preis, 2011 [36]	Neural-network support for liver lesion interpretation	98 consecutive patients	PET with MRI comparator	Neural network	Expert MRI interpretation	NN AUC 0.905 and 0.896; readers 0.786 and 0.796; assisted readers 0.924 and 0.881	No	Not HCC-specific; PET adjunct study rather than surveillance or dedicated HCC diagnosis
Mokrane, 2020 [15]	HCC diagnosis in indeterminate cirrhotic nodules	178 total; 142 discovery/36 validation	Triphasic CT radiomics	Radiomics ML signature	Biopsy-proven HCC vs. non-HCC	AUC 0.70 discovery; 0.66 validation	Yes	Small validation cohort; manual segmentation; difficult indeterminate-nodule setting
Yasaka, 2018 [37]	Liver mass differentiation on dynamic CT	460 training/100 temporal test image sets	Dynamic contrast CT	CNN	Radiologic lesion category assignment	Median accuracy 0.84; median AUC 0.92	Temporal test set only	Single-center preliminary study with augmented images and typical lesions
Shi, 2020 [38]	HCC vs. non-HCC focal liver lesion differentiation	342 patients/449 lesions (internal holdout test)	Four-phase and three-phase CT	Convolutional dense networks	Lesion diagnosis	Accuracy 83.3%, 81.1%, 85.6%; AUC 0.925, 0.862, 0.920	No	Internal validation only; lesion-enriched multiclass dataset
Wang, 2021 [16]	HCC diagnosis from CT	7512 train/385 internal test/556 external test	CT imaging	Deep-learning system	HCC case classification	Internal AUROC 0.887, sensitivity 78.4%, specificity 84.4%; external AUROC 0.883, sensitivity 89.4%, specificity 74.0%	Yes	Retrospective diagnostic CT cohort; protocol and case-mix shift remain concerns
Oyama, 2019 [39]	MRI-based hepatic tumor classification	150 tumors total	Non-contrast 3D T1 MRI	Texture/topology radiomics ML	Tumor class assignment	Accuracy 92% for HCC vs. metastasis; 90% for HCC vs. hemangioma	No	Balanced retrospective dataset; not a surveillance or cirrhosis-enriched workflow
Oestmann, 2021 [17]	Typical/atypical HCC vs. non-HCC on MRI	150 lesions total	Contrast-enhanced MRI	3D CNN	Pathology-proven lesion diagnosis	Accuracy 87.3%; sensitivity 92.7%; specificity 82.0%; AUC 0.912	No	Internal resampling rather than geographic external validation; atypical-lesion performance lower
Zhen, 2025 [18]	Liver tumor classification on non-contrast MRI	1959 patients; 50,418 dev images/5172 internal/2916 external-1/1338 external-2	Non-contrast MRI	Inception-ResNet V2	Tumor diagnosis	Internal AUC 0.91/0.873/0.876 across classes; malignant-tumor sensitivity 98.1%, 89.7%, 87.5%	Yes	Mixed liver tumors rather than HCC-only; image-level validation across heterogeneous scanners
Hamm, 2019 [40]	Classification of hepatic lesions on multiphasic MRI	494 lesions; 434 train/60 test	Multiphasic MRI	CNN	Lesion diagnosis across six categories	Accuracy 92%; sensitivity 92%; specificity 98%	No	Single-center proof-of-concept with typical lesions
Atupelage, 2013 [41]	Computational HCC grading	109 patients/369 ROI images	Histopathology ROIs	Bag-of-features + multifractal descriptor	Edmondson-Steiner grading	Average correct classification ≈95%	No	ROI-based handcrafted-feature pipeline; no slide-level or external testing
Atupelage, 2014 [42]	Computational HCC grading from nuclei	1120 subimages derived from WSI ROIs	Histopathology ROIs/subimages	Cell-nuclei classification pipeline	Edmondson-Steiner grading	Correct classification rate 95.97%	No	Subimage-based evaluation with handcrafted nuclei features; no external validation
Kiani, 2020 [43]	Histopathologic HCC vs. CCA with AI assistance	26 validation WSI/80 independent test WSI	H&E whole-slide images	Deep-learning assistant	Pathologist-labeled HCC vs. cholangiocarcinoma	Model accuracy 0.885 validation; 0.842 independent test; assisted OR 1.499 in defined subgroup	Yes	Single-center binary task; assistance could reduce pathologist accuracy when the model erred
Liao, 2020 [44]	Histopathology classification and prognosis	481 TCGA WSIs/719 WCH TMA dots§	H&E histopathology	Quantitative image features + ML/RF prognostic model	HCC vs. adjacent normal tissue; survival after resection	Diagnostic AUC 0.988 test; 0.886 external validation	Yes	Retrospective slide-based study; handcrafted features and cohort-shift risk between WSI and TMA
Wang R, 2020 [45]	Hyperspectral pathology classification	HCC sample-slice dataset	Hyperspectral pathology data	1-D CNN	Tumor-tissue classification	Sensitivity 0.871; specificity 0.888; AUC 0.950	No	Specialized hyperspectral platform with uncertain routine-pathology portability
Lin, 2019 [46]	HCC differentiation grading	217 microscopy images	Multiphoton microscopy images	VGG-16 CNN	Histologic differentiation grade	Classification accuracy >90%	No	Specialized microscopy workflow; image-level dataset rather than routine pathology workflow
Chen, 2020 [47]	Histopathology classification + mutation prediction	481 TCGA WSIs/719 WCH TMA dots	H&E histopathology	Inception V3	Diagnosis, differentiation, and mutation status	Accuracy 96.0% benign vs. malignant; 89.6% differentiation; external mutation AUC 0.71–0.89	Yes	Curated retrospective slide cohorts; mutation prediction limited to selected genes
Wang H, 2020 [48]	Spatial pathology subtype discovery	66 patients for training/testing nuclei labels; 304 TCGA tumors for application	H&E whole-slide images	Mask R-CNN + clustering	Single-cell segmentation/classification and survival-linked subtype discovery	Nuclei detection accuracy 92%; tumor-cell classification 98%; lymphocyte classification 91% in testing	No external clinical validation	Subtype-discovery study rather than direct diagnostic deployment; TCGA-only downstream cohort
Abajian, 2018 [21]	Response prediction before intra-arterial therapy	36 patients	MRI + clinical data	Logistic regression/random forest	qEASL treatment response	Accuracy 78%; sensitivity 62.5%; specificity 82.1%	No	Very small retrospective cohort; mixed embolic techniques
Morshid, 2019 [49]	Response prediction to first TACE	105 patients	CT + clinical variables	Random forest	mRECIST-based TTP cutoff (14 weeks)	Accuracy 74.2% vs. 62.9% for BCLC alone; ROC AUC 0.73	No	Single-center retrospective design with surrogate TTP dichotomization
Liu, 2020 [22]	Response prediction to first TACE	89 training/41 validation	CEUS cines	Deep-learning radiomics	mRECIST objective response after first TACE	Validation AUC 0.93	No	Single-center retrospective CEUS cohort; response surrogate rather than survival
Peng, 2020 [23]	Response prediction to TACE	562 development/89 external-val-1/138 external-val-2	Pretreatment CT	ResNet50	CR/PR/SD/PD after TACE	Accuracy 84.3%; AUC 0.97/0.96/0.95/0.96; external accuracies 85.1% and 82.8%	Yes	Retrospective multicenter Chinese cohorts; patch-level prediction with uncertain calibration
Dai, 2025 [24]	Prognosis for repeat TACE	310 patients across 3 hospitals	CT radiomics + DL + HBsAg	Deep-learning radiomics score	Prognosis after repeat TACE	AUCs ≈0.97 development, 0.89 validation, 0.76 and 0.84 external cohorts	Yes	Restricted to repeat-TACE population; retrospective multicenter design
Mähringer-Kunz, 2020 [25]	Survival prediction after TACE	282 total; 225 train/57 validation	Clinical variables	Artificial neural network	1-year overall survival after TACE	Validation AUC 0.83 ± 0.06; sensitivity 77.8%; specificity 81.0%	No	Single-center pilot study without external validation
Ibragimov, 2018 [50]	Hepatobiliary toxicity prediction after liver SBRT	125 total (36 HCC, 58 metastases, 27 CCA, 4 other)	3D dose plan + clinical variables	CNN + fully connected network	Hepatobiliary toxicity after SBRT	CNN AUC 0.79; combined model AUC 0.85	No	Mixed liver histologies; toxicity endpoint rather than tumor control
Wu, 2017 [29]	DFS prediction after RFA	252 for 1-year DFS; 179 for 2-year DFS	Clinical variables	Artificial neural network	1-year and 2-year DFS after RFA	1-year AUC 0.84 internal/0.77 simulated prospective; 2-year AUC 0.75/0.72	No	Single-center retrospective study with simulated rather than true prospective validation
Saillard, 2020 [27]	Survival prediction after resection	194 discovery/328 independent validation	Histologic whole-slide images	SCHMOWDER/CHOWDER deep learning	Overall survival after resection	C-index 0.78 and 0.75 in discovery; external validation retained superiority vs. baseline score	Yes	Retrospective slide-based cohorts; calibration and clinical action thresholds not fully established
Zhou, 2019 [51]	Survival prediction by machine-learning classification rules	165 cases	Clinical survival dataset	Machine-learning classification rules	Overall survival/survival class	Conference abstract; detailed metrics not fully reported beyond survival-prediction framing	No public external validation	Conference abstract with sparse methodological detail
Ji, 2019 [26]	Recurrence prediction after curative resection	210 train/107 internal/153 external	Contrast-enhanced CT radiomics + clinical data	Aggregated ML radiomics + Cox models	Recurrence-free survival after resection	Radiomics signature C-index 0.633–0.699; combined model 0.733–0.801; IBS 0.147–0.165	Yes	Retrospective manual/semimanual segmentation; solitary-HCC surgical cohort only
Wang W, 2019 [52]	Early recurrence prediction after resection	167 cases/765 labeled slices	Multi-phase CT + clinical data	Deep-learning radiomics	Early recurrence within 1 year after resection	AUC 0.825	Internal cross-validation only	Conference-paper model with limited reporting and no geographic external validation
Xu, 2019 [53]	Early vs. late vs. no recurrence prediction	Real-world HCC cohort + separate out-of-sample dataset	Clinical variables with a latent-variable Bayesian network	Bayesian network with a latent variable	Early, late, or no recurrence after resection	Outperformed three benchmark techniques on accuracy, precision, recall, and F-measure	Yes, out-of-sample dataset	Exact cohort split not transparently reported; retrospective clinical data
Feng, 2019 [19]	Preoperative microvascular invasion prediction	110 training/50 validation	Gd-EOB-DTPA MRI radiomics	Radiomics + supervised ML	Histologic microvascular invasion	Training AUC 0.85; validation AUC 0.83; validation sensitivity 90.0%; specificity 75.0%	No	Single-center manual-VOI radiomics study using a surrogate biologic endpoint
Tsilimigras, 2020 [20]	Pre/postoperative assessment beyond BCLC resection criteria	976 patients	Clinical + pathologic variables	CART	Overall survival after resection	5-year OS 64.2% for BCLC 0/A vs. 50.2% for BCLC B; CART identified stage-specific key variables	No	Retrospective surgery-only cohort; rule-based partitioning may be unstable across cohorts
Guo, 2019 [28]	Recurrence prediction after liver transplantation	93 training/40 validation	Pretransplant CT radiomics + clinical variables	LASSO-Cox radiomics nomogram	Recurrence-free survival after liver transplantation	Combined-model C-index 0.785 training; 0.789 validation; calibration *p* = 0.121 and 0.164	No true external site validation	Small retrospective transplant cohort with center-specific selection practices

Abbreviations: AFP, alpha-fetoprotein; ANN, artificial neural network; AUC/AUROC, area under the receiver operating characteristic curve; BCLC, Barcelona Clinic Liver Cancer; CAD, computer-aided diagnosis; CART, classification and regression tree; CEUS, contrast-enhanced ultrasound; CNN, convolutional neural network; DFS, disease-free survival; DNN, deep neural network; HBsAg, hepatitis B surface antigen; HCC, hepatocellular carcinoma; LR-M, probably or definitely malignant but not specific for HCC; mRECIST, modified Response Evaluation Criteria in Solid Tumors; MRI, magnetic resonance imaging; PET, positron emission tomography; qEASL, quantitative European Association for the Study of the Liver; RFA, radiofrequency ablation; ROI, region of interest; SBRT, stereotactic body radiotherapy; TCGA, The Cancer Genome Atlas; TMA, tissue microarray; TTP, time to progression; US, ultrasound; VHA, Veterans Health Administration; WSI, whole-slide image. Dataset size is reported as development and validation when those splits were available in the source. Reference standard is shown explicitly because outcomes ranged from pathology and imaging-based diagnosis to treatment response and time-to-event endpoints; performance metrics are therefore not directly comparable across rows. Most diagnostic imaging studies were performed in retrospective lesion-enriched datasets rather than true surveillance populations, and most prognostic models remain retrospective despite improving external validation.

**Table 3:** Common sources of bias and the minimum reporting expectations for future HCC AI studies. Each row identifies a methodological problem recurring across the HCC AI literature, explains why it threatens the clinical validity or transportability of reported findings, and specifies the minimum information future studies should provide to allow meaningful appraisal. Items are drawn from STARD-AI, TRIPOD + AI, PROBAST + AI, and FUTURE-AI reporting frameworks and are intended as a practical checklist for authors, peer reviewers, and journal editors evaluating HCC AI manuscripts.

Problem	Why It Matters	What Future Studies Should Report
Lesion-enriched retrospective datasets	Inflates discrimination and does not reflect surveillance prevalence or image quality	Intended use population, prevalence, case mix, and whether the cohort is surveillance-like or diagnostic
Internal validation only	Does not establish transportability across sites or time periods	Temporal and external validation, ideally across multiple institutions
Heterogeneous reference standards	Makes apparent accuracy difficult to compare across studies	Clear description of pathology, imaging, or clinical adjudication used as ground truth
Poor calibration reporting	A high AUROC can still yield unsafe absolute risk estimates	Calibration plots, calibration slope/intercept, and clinically meaningful thresholds
Radiomics instability	Scanner, reconstruction, and segmentation choices alter features	Acquisition details, preprocessing pipeline, IBSI-compliant feature definitions, reproducibility checks
Human-factors blind spots	A good algorithm can still fail when badly integrated into the workflow	User role, alert logic, interface design, override behavior, and silent-mode evaluation

Abbreviations: AI, artificial intelligence; AUROC, area under the receiver operating characteristic curve; HCC, hepatocellular carcinoma; IBSI, Image Biomarker Standardisation Initiative.

## Data Availability

Not applicable.

## Abbreviations

AFP, alpha-fetoprotein; AI, artificial intelligence; ANN, artificial neural network; ART, Assessment for Retreatment with TACE score; AASLD, American Association for the Study of Liver Diseases; ABCR, ABCR score; APASL, Asian Pacific Association for the Study of the Liver; AUC, area under the curve; AUROC, area under the receiver operating characteristic curve; BCLC, Barcelona Clinic Liver Cancer; c-CCA, combined hepatocellular-cholangiocarcinoma; CEUS, contrast-enhanced ultrasound; CI, confidence interval; CNN, convolutional neural network; CT, computed tomography; DCNN/DCNNs, deep convolutional neural network(s); DFS, disease-free survival; DL, deep learning; DVH, dose–volume histogram; ECG, electrocardiogram; EASL, European Association for the Study of the Liver; EBioMedicine, EBioMedicine (journal); EHR, electronic health record; ER, emergency room; FDA, U.S. Food and Drug Administration; H&E, hematoxylin and eosin; HCC, hepatocellular carcinoma; i-CCA, intrahepatic cholangiocarcinoma; IPD, individual-participant data; LI-RADS, Liver Imaging Reporting and Data System; LiTS, Liver Tumor Segmentation (challenge); MELD, Model for End-Stage Liver Disease; ML, machine learning; mRECIST, modified Response Evaluation Criteria in Solid Tumors; MRI/MR, magnetic resonance imaging; MPM, multiphoton microscopy; NAFLD, nonalcoholic fatty liver disease; NASH, nonalcoholic steatohepatitis; PFS, progression-free survival; RFA, radiofrequency ablation; SBRT, stereotactic body radiation therapy; SNACOR, SNACOR score; STARD, Standards for Reporting of Diagnostic Accuracy Studies; STARD-AI, STARD extension for artificial intelligence; TACE, transarterial chemoembolization; TARE, transarterial radioembolization; TTP, time to progression; UCSF, University of California, San Francisco.
